# IRv2-Net: A Deep Learning Framework for Enhanced Polyp Segmentation Performance Integrating InceptionResNetV2 and UNet Architecture with Test Time Augmentation Techniques

**DOI:** 10.3390/s23187724

**Published:** 2023-09-07

**Authors:** Md. Faysal Ahamed, Md. Khalid Syfullah, Ovi Sarkar, Md. Tohidul Islam, Md. Nahiduzzaman, Md. Rabiul Islam, Amith Khandakar, Mohamed Arselene Ayari, Muhammad E. H. Chowdhury

**Affiliations:** 1 Department of Computer Science & Engineering, Rajshahi University of Engineering & Technology, Rajshahi 6204, Bangladesh; faysalahamedjishan@gmail.com (M.F.A.); rabiul.cse@gmail.com (M.R.I.); 2 Department of Electrical & Computer Engineering, Rajshahi University of Engineering & Technology, Rajshahi 6204, Bangladesh; khalidsyfullah@gmail.com (M.K.S.); ovisarkareceian@gmail.com (O.S.); nahiduzzaman@ece.ruet.ac.bd (M.N.); 3 Department of Information & Communication Engineering, University of Rajshahi, Rajshahi 6205, Bangladesh; tanzid1971@gmail.com; 4 Department of Electrical Engineering, Qatar University, Doha 2713, Qatar; amitk@qu.edu.qa; 5 Department of Civil and environmental Engineering, Qatar University, Doha 2713, Qatar; arslana@qu.edu.qa; 6 Technology Innovation and Engineering Education Unit (TIEE), Qatar University, Doha 2713, Qatar

**Keywords:** segmentation, colonoscopy, IRv2-Net, Kvasir-SEG, CVC-ClinicDB, test time augmentation, polyps

## Abstract

Colorectal polyps in the colon or rectum are precancerous growths that can lead to a more severe disease called colorectal cancer. Accurate segmentation of polyps using medical imaging data is essential for effective diagnosis. However, manual segmentation by endoscopists can be time-consuming, error-prone, and expensive, leading to a high rate of missed anomalies. To solve this problem, an automated diagnostic system based on deep learning algorithms is proposed to find polyps. The proposed IRv2-Net model is developed using the UNet architecture with a pre-trained InceptionResNetV2 encoder to extract most features from the input samples. The Test Time Augmentation (TTA) technique, which utilizes the characteristics of the original, horizontal, and vertical flips, is used to gain precise boundary information and multi-scale image features. The performance of numerous state-of-the-art (SOTA) models is compared using several metrics such as accuracy, Dice Similarity Coefficients (DSC), Intersection Over Union (IoU), precision, and recall. The proposed model is tested on the Kvasir-SEG and CVC-ClinicDB datasets, demonstrating superior performance in handling unseen real-time data. It achieves the highest area coverage in the area under the Receiver Operating Characteristic (ROC-AUC) and area under Precision-Recall (AUC-PR) curves. The model exhibits excellent qualitative testing outcomes across different types of polyps, including more oversized, smaller, over-saturated, sessile, or flat polyps, within the same dataset and across different datasets. Our approach can significantly minimize the number of missed rating difficulties. Lastly, a graphical interface is developed for producing the mask in real-time. The findings of this study have potential applications in clinical colonoscopy procedures and can serve based on further research and development.

## 1. Introduction

**Figure 1:**
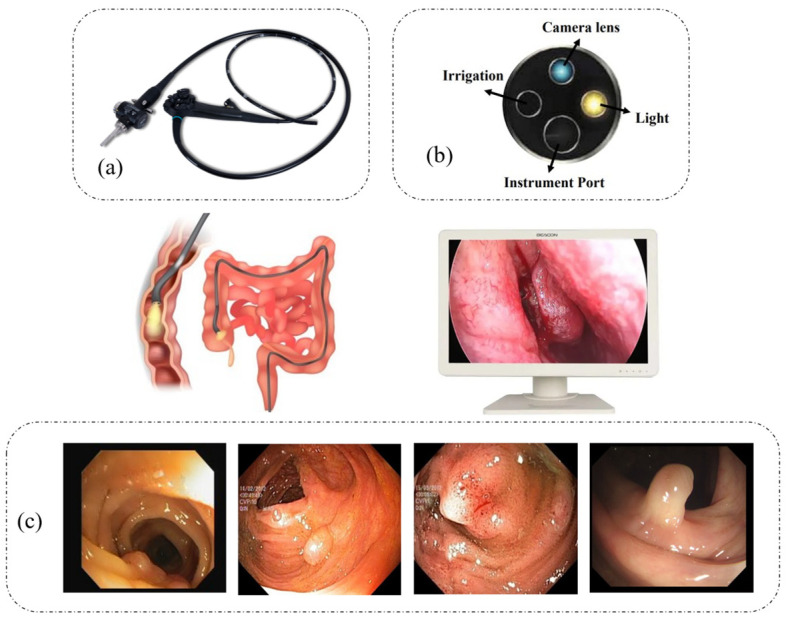
A visual representation of a colonoscopy includes (**a**) an example of the endoscope, (**b**) an endoscope probe, which is inserted into the body to furnish endoscopic images, and (**c**) several examples of colorectal polyp images obtained during the colonoscopy procedure.

One of the primary causes of death from cancer worldwide is colorectal cancer (CRC) [1]. Colon cancer comprises 63% of all cancer cases worldwide, whereas stomach cancer accounts for 33%. Stage IV colon cancer is the most dangerous. Stage I colon cancer has a 95% five-year survival rate, but stage IV colon cancer has a 3% survival rate [2]. This demonstrates how crucial early cancer detection is. A colonoscopy is one of the colon cancer screening procedures that can help detect the condition early [3]. The scope of this medical operation extends to the rectum and colon. This makes any inflammatory tissue, ulcers, or abnormal growths visible to doctors [4]. Gastroenterologists strongly support colonoscopy screening because it enables an endoscopist to carefully examine and monitor the colon using a flexible camera (see Figure 1). This approach is superior to other colon surveillance options since polyps may be detected and removed promptly.

Polyps are abnormal tissue growths that can arise in the digestive system and need to be recognized to prevent the development of advanced stages of colorectal cancer. They are primarily found in the colorectal region of the Gastrointestinal tract. They are usually recognized as precursors to CRC. Colorectal polyps can be divided into three sizes: diminutive (≤5 mm), small (6 to 9 mm), and advanced or extensive (≥10 mm) [5]. Larger polyps typically show more symptoms and are more likely to be found during a colonoscopy. Minor polyps can be removed with a little snare during the process, while larger polyps may need surgical intervention. Small and insignificant colorectal polyps constitute a significant source of colorectal cancer risk. Since there is minimal chance of injuring the surrounding tissues, the cold snare polypectomy is the most successful technique for removing tiny polyps [6]. This factor is crucial since it lowers the likelihood of problems like bleeding and infection.

While colonoscopy is a successful method for finding and removing polyps, it has several drawbacks. There is a chance that polyps may be missed, which could lead to a higher risk of cancer [7]. While newer instruments and techniques facilitate polyp detection, some cases still go unnoticed. Polyps can vary in size, shape (small, large, flat, or elliptical), and growth stage, making accurate differentiation challenging as they can easily be confused with the surrounding mucous. Most individuals have adenomas, serrated polyps, hyperplastic polyps, or mixed polyps. These different types of polyps can resemble one another, and even within the same type, significant variations can exist. Furthermore, identifying polyps from background objects, such as surrounding tissue, might sometimes be challenging. Obtaining colonoscopy videos that were evaluated by endoscopists in clinical facilities can be time-consuming [8]. This could lead to missed early stage detections.

There is a need for quicker and more precise analytic procedures because colonoscopy data interpretation is labor-intensive, subjective, and challenging. Artificial intelligence (AI) integration has become a viable method for boosting colonoscopy analysis and segmentation in recent years, potentially increasing diagnostic accuracy and accelerating patient care. Deep learning-based systems show remarkable performance in automatically spotting and segmenting polyps in colonoscopy images, as well as video [9]. In principle, these Computer-Aided Detection (CAD) systems should possess the following characteristics:

- They should display trustworthy results by displaying consistent performance and improving adaptability based on patient differences.
- They should outperform the established benchmarks for algorithms and attain excellent overall performance.
- The capacity to function in real-time is essential for clinical application.
- The systems should be simple to use and offer results that medical practitioners may quickly understand.
- Correspondingly, making these processes work for a large group of people is both cost and resource effective.

In the first step, the focus is on finding, locating, and dividing colorectal polyps, which are known to be early indicators of colorectal cancer (CRC). This is because a lot of colon cancers start in benign adenomatous polyps with abnormal cell composition. To stop cancer from spreading, these polyps can be identified and removed. The ability of an endoscopist to detect polyps has a strong influence on the possibility of developing CRC within 60 months of a colonoscopy [10].

Polyp identification and localization are essential to count the number of polyps in a patient and conduct routine surveillance. Pixel-wise segmentation is crucial for the automated labeling of polyp margins during surgery or radio-frequency ablation. Recent advances in semantic segmentation-based algorithms for medical image analysis are investigated to improve the efficiency of automated polyp segmentation and identification [11]. This study suggests that the proposed IRv2-Net model (UNet architecture with a pre-train InceptionResNetV2 encoder) precisely divides polyp locations and separates them from the surrounding tissues. The model is assessed using two publicly accessible datasets, and experimental findings show that it performs more effectively and efficiently than well-known architectures like UNet [12], Attention-UNet [13], ResUNet [14], and ResUNet++ [15]. This work made the following contributions, which are summarized below:

1. An encoder–decoder architecture, IRv2-Net, is developed specifically for colonoscopy image segmentation. This architecture stands out for its high-detection rate, allowing real-time polyp segmentation, while maintaining a competitive level of accuracy.
2. To compare the results, the Kvasir-SEG and CVC-ClinicDB databases are employed. To improve the model’s learning efficacy, 26 different types of image pre-processing are applied. The IRv2-Net model is better at detecting small, flat, sessile, and over-saturated polyps that are frequently missed during a colonoscopy examination.
3. Test time Augmentation (TTA) techniques are integrated with the IRv2-Net model to improve the overall segmentation performance. From the original, horizontal and vertical flips are averaged to enhance the segmented prediction more accurately.
4. We designed a Graphical User Interface (GUI) as a CAD system to instantaneously predict the polyp mask from the input sample, which makes the process of diagnosis more precise and straightforward.
5. Our main goal is to make a deep-learning model that can be used more often in clinical applications. This is why we trained our model on Kvasir-SEG data and tested it on CVC-ClinicDB data and vice versa. Our model performs better than others on unseen data collected in different places and devices.

The remaining part is divided into the following sections: a summary of several recent works in this field is provided in Section 2; Section 3 discusses the suggested architecture and experimental materials; Section 4 offers an experimental setup with performance metrics; Section 5 presents performance analysis and evaluation while showcasing the experiment’s findings; and Section 6 outlines this work’s conclusions and future directions.

## 2. Related Works

Researchers have been working on CAD prototypes that can automatically segment polyps. The majority of early methods for segmenting polyps depended on analyzing the polyp’s edge. However, contemporary methods increasingly make use of CNN and pre-trained networks. The Kvasir-SEG dataset [15,16,17,18,19,20,21,22,23], the CVC-ClinicDB dataset [24,25,26,27,28,29,30], and the TTA [31,32,33,34,35,36,37] are frequently used datasets in analyses of polyp detection and segmentation networks. 

The ResUNet++ [15] architecture for segmenting colonoscopy images achieved high evaluation scores with DSC of 81.33% and an IoU of 79.27% on the Kvasir-SEG, and DSC of 79.55% and an IoU of 79.62% on the CVC-612 dataset, respectively. Jha et al. [16] introduced NanoNet, which outperformed more complex models regarding segmentation accuracy while maintaining compact parameters. But this model achieved a lesser IoU of 0.7282. Another study, by Valanarasu et al. [17] proposed a novel model UNeXt network that combined CLs with tokenized MLP blocks in the latent space; combining these elements and the channel shifting technique resulted in better image segmentation performance with a 0.8843 recall but a lower IoU score of 0.6284. Likewise, Jha et al. [18] introduced TransNetR, an encoder–decoder architecture featuring three decoder blocks and a concluding upsampling layer. When evaluated on the Kvasir-SEG dataset, TransNetR demonstrated remarkable performance, attaining a substantial DSC of 0.8706 and a mean IoU of 0.8016, recall of 0.8843, and precision of 0.9073. Impressively, it achieved a processing speed of 54.60, outperforming its predecessor. Wen et al. [19] devised a highly effective solution to tackle overfitting by combining FCN and CNN. Their revolutionary segmentation pipeline training method included an interactive weight transfer technique, which proved far superior to other approaches, delivering a remarkable DSC score of 0.8922.

Yue et al. [20] proposed the attention-guided pyramid context network APCNet. Their proposed network resolved existing approaches’ shortcomings by incorporating feature interaction across multiple layers and utilizing an attention-guided multilayer aggregation mechanism. Similar to Hong et al. [21], Wang et al. [22] focused on the mask region’s bounding box to extract additional multilayer information. ColonFormer is an innovative encoder–decoder architecture devised by Duc et al. [23]. Both the encoder and decoder branches captured long-range semantic information. The encoder models global semantic relations at multiple scales using transformers, whereas the decoder learns multi-level features for improved representation utilizing a hierarchical network structure. Implementing a refinement module with a skip connection technique improves the accuracy of polyp border segmentation.

Sasmal et al. [24] described a real-time monitoring method for segmenting polyp regions in colonoscopy video frames. The system used a modified tracking technique and a saliency map to locate the polyp region. The measurement model consisted of a probabilistic visual saliency map depicting polyp characteristics. A dynamic contour model based on the elliptical shape of the polyps improved the localization. The proposed technique obtained an average Dice score of 66.06% in CVC-ClinicDB, highlighting its potential for polyp localization and diagnosis. Guo et al. [25] combined the deep residual network and dilation kernel layers within the FCNN framework, and the U-net network was enhanced with dilation kernels merging “squeeze and extraction” units having a Dice value of 0.89. 

In their research, Lou et al. [26] introduced CaraNet, a model designed to improve the segmentation of small medical objects by incorporating context axial reverse attention and channel-wise feature pyramid modules. The model’s effectiveness was thoroughly assessed across diverse polyp segmentation datasets, revealing outstanding outcomes. Notably, on the CVC-ClinicDB dataset, CaraNet achieved remarkable metrics of 0.936 (DSC) and 0.887 (IoU).

To solve the vanishing gradient problem and provide contextual data, Vahid et al. [27] suggested a Res-UNet architectural model that used successive networks with multi-scale attention gates, residual blocks, and skip connections. The CVC-ClinicDB dataset showed remarkable segmentation findings with a Dice of 83% and Jaccard of 75.31%. In the research they examined, John et al. [28] developed the Polyp Segmentation Network (PSNet), which employed a dual network architecture and incorporated multiple deep learning modules. This approach addressed overfitting and boundary definition issues, leading to remarkable outcomes on CVC-ClinicDB dataset. Specifically, the PSNet achieved Dice and IoU scores of 0.928 and 0.879, respectively, indicating its superior performance in polyp segmentation. A CNN architecture was introduced by Razvan et al. [29] that effectively captures information at varying resolutions using a custom convolutional block and residual downsampling. It is essential to note that the network is under strict supervision. To extract feature maps from the colorectal images, Nguyen et al. [30] developed a deep network with Random Changing Pixel Value Augmentation that employed Deeplabv3 as an encoder. The Otsu thresholding technique converts the FCN probability map into a binary image, from which the most significant connected component is extracted and used to determine the most likely location of a polyp. Their proposed Deep Encoder–Decoder Network achieved a Dice score of 88.9% or higher on the CVC-ClinicDB.

Recent research by Muller et al. [31] devised a novel classification method for medical images. This strategy employed ensemble learning techniques, incorporating preprocessing techniques, image enhancement strategies, and nine, deep CNN architectures. Extensive testing on four different datasets confirmed that stacking was the most effective method with improvements to the F1-score metric of up to 13%. Augmenting resulted in a 4% enhancement in single model-based pipelines, whereas Bagging increased the F1 score by 11%. 

During the testing phase, the technique of test time augmentation can have a significant impact on performance [32]. By dynamically applying augmentation strategies, the model’s generalization and robustness can be enhanced [33]. Calvo-Zaragoza et al. [34] examined the efficacy of data augmentation in test samples utilizing ensemble predictions. Even within the limited data margin, this method enhanced performance significantly. The results of the experimental research demonstrated that the aggregation of augmentations in test samples effectively handles the limitations associated with correct prediction [35]. Ayhan et al. [36] observed that accurately estimating uncertainty levels for the model’s output decisions was challenging. However, this issue could be reduced by using augmentation with the tested samples to assess the network output variability. Kandel et al. [37] conducted a study on the MURA dataset to examine the impact of TTA. They assessed the effectiveness of nine different augmentation methods compared to predictions made without TTA. The findings revealed that TTA significantly enhances the accuracy of medical images. 

The cross-dataset test was unable to conclusively determine whether the CAD system was generalizable or not in the aforementioned studies’ primary limitation. Creating a universal model reliably and efficiently segmenting polyps from various sources was crucial to advancing CAD for automated segmentation. No postprocessing techniques were used to obtain a more accurate, real-time polyp region. In order to obtain the best results, several lengthy experiments required a large number of colonoscopy images.

## 3. Methodology

**Figure 2:**
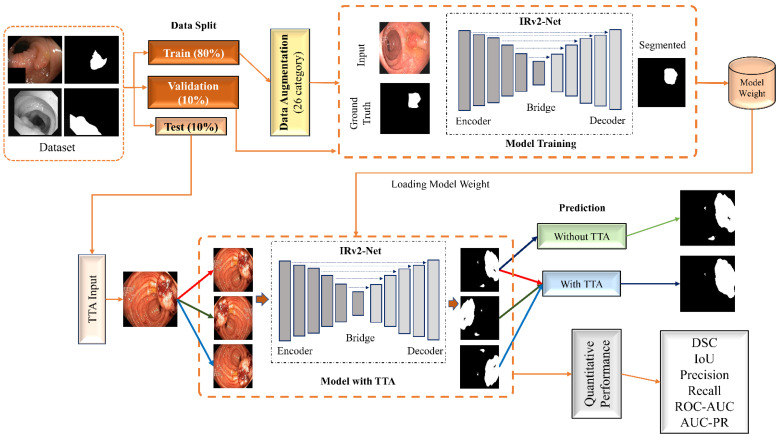
A visual representation of proposed research framework for segmenting the polyp regions. Dataset splitting into three categories (80% Training, 10% Validation, 10% Testing). Training and data pass through Data Augmentation before model training and the model is validated on validation data during model training. Final prediction is done with/without TTA Augmentation. Where original image is used to generate mask prediction without TTA and H-flipped, V-flipped images are used to generate mask prediction with TTA respectively. Finally, Quantitative analysis is performed on six different metrics.

Figure 2 visually depicts the research methodology used in this study where orange arrow denotes the flow of working steps. We used the Kvasir-SEG and CVC-ClinicDB datasets to train the model, dividing each into separate subsets for training, validation, and testing. We only used augmented samples for training, and we carefully preserved the model weights for subsequent evaluations. We reinforced the integrity of our training data through a meticulous verification process, which involved assessing and validating the accuracy of the ground truth annotations for each image. We integrated TTA techniques into the model to visualize the improved segmentation performance, utilizing multiple predictions created from test set images through the model weights where red, green, and blue arrow define original image, horizontal, and vertical image processing flow (see Figure 2). These predictions were averaged to produce an improved segmented outcome. We assessed performance metrics quantitatively, including Dice Similarity Coefficients (DSC), Intersection Over Union (IoU), precision, recall, Receiver Operating Characteristic (ROC-AUC) and area under Precision-Recall (AUC-PR) curves. Furthermore, qualitative evaluations were conducted to assess the visual and accuracy aspects of the segmentation outcomes.

For model training, we considered UNet, Attention-UNet, ResUNet, ResUNet++, and the proposed IRv2-Net. The main significant differences among these models are as follows: UNet employs a basic encoder–decoder structure with skip connections, excelling at capturing context and spatial information using convolutional layers (CLs), Maxpooling (MP), rectified linear units (ReLU), and transposed CLs. Attention-UNet introduces a noteworthy improvement through its attention mechanism, enabling the network to focus on relevant features and suppress noise. ResUNet incorporates a residual path into its architecture for complex feature extraction, providing shortcuts for gradient propagation from output to earlier layers, allowing the network to learn both original and residual features separately. ResUNet++ further enhances the model by integrating Squeeze-and-Excitation (SE) blocks and Atrous Spatial Pyramid Pooling (ASPP) techniques. SE blocks highlight relevant spatial and channel-wise features while reducing the impact of less discriminative ones, while ASPP employs different dilation rates (atrous rates) to capture multi-scale contextual information from the input. In contrast, the proposed IRv2-Net leverages the Inception-ResNet-v2 architecture, enabling it to effectively capture fine details, contextual information, and multi-scale features. By integrating UNet’s skip connection mechanism, IRv2-Net refines the seg-mentation map with both low-level and high-level features, ensuring accurate capture of intricate structures like polyps. This overcomes limitations in traditional UNet-based models like Attention-UNet that might lose fine-grained information.

### 3.1. Dataset Description

**Figure 3:**
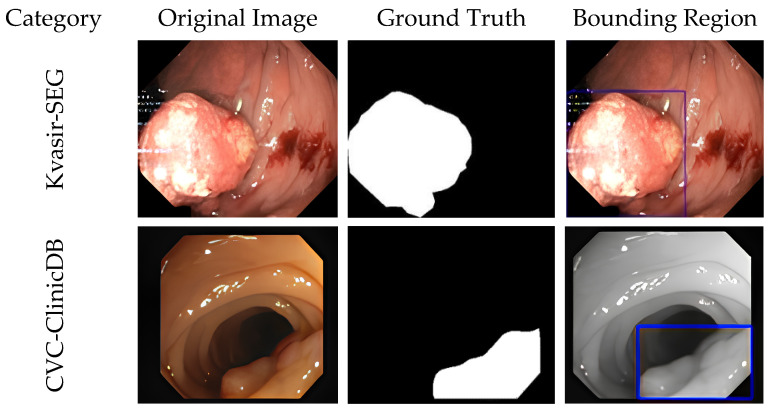
Samples on both Kvasir-SEG and CVC-ClinicDB datasets are presented with ground truth and bounded box. Bounded rectangular blue and purple boxes denote the region of colorectal polyp.

The Kvasir-SEG and CVC-ClinicDB datasets were utilized in this research. Figure 3 presents both Kvasir-SEG and CVC-ClinicDB datasets, which represent the patient’s colorectal organ [38]. Those high-resolution images were collected using an electromagnetic imaging system. Kvasir-SEG images have resolutions ranging from 332 × 487 to 1920 × 1072 pixels, 96 dpi, and a 24-bit depth. The dataset consists of a total of 1000 images, where there are 700 large, 252 medium, and 48 small polyps. The large category polyps have a pixel count > 160 × 160, medium polyps > 64 × 64, and small polyps < 63 × 63. The dataset (https://datasets.simula.no/kvasir-seg/ (accessed on 10 May 2023)) was annotated by Oslo University in Norway. The KVASIR-SEG dataset annotation process involved multiple steps. It was annotated by two expert gastroenterologists who were trained to identify and classify different types of lesions. Additionally, a third expert was consulted to resolve any disagreements. CVC-ClinicDB includes 612 TIFF-formatted images [1]. Each image has dimensions of 288 × 384 pixels, a resolution of 96 dpi, and a 24-bit depth. The CVC-ClinicDB2 dataset was extracted from colonoscopy videos and served as the official training database for the 2015 MICCAI Sub-Challenge on Automatic Polyp Detection Challenge. The CVC-ClinicDB data was annotated by expert video endoscopists affiliated with the clinical Hospitals in Barcelona, Spain, who delineated the exact boundaries of any polyps identified from 31 video sequence and 23 patients. The binary mask was obtained through a careful outlining procedure conducted by a certified specialist from each facility.

### 3.2. Pre-Processing

In medical imaging, data augmentation is essential for increasing the size of existing samples. These techniques create synthetic variations of existing samples. To guarantee the model was trained in a variety of ways, the polyp datasets were split 80:10:10 between training, validation, and testing sets. Twenty-six preprocessing methods were used, each adding unique variations to enhance diversity and robustness.

**Figure 4:**
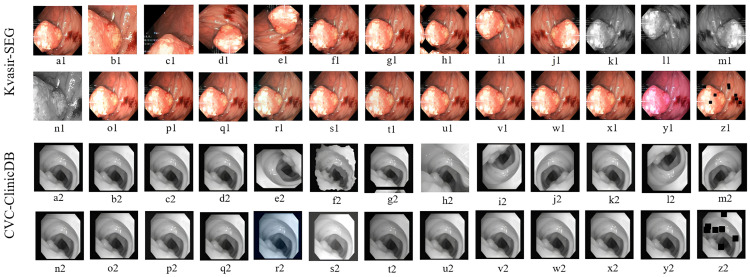
An illustration of all pre-processing, which include (**a1**,**a2**) center crop, (**b1**,**b2**) crop, (**c1**,**c2**) random crop, (**d1**,**d2**) random 90-degree rotation, (**e1**,**e2**) transpose, (**f1**,**f2**) elastic transformation, (**g1**,**g2**) grid distortion, (**h1**,**h2**) optical distortion, (**i1**,**i2**) vertical flip, (**j1**,**j2**) horizontal flip, (**k1**,**k2**) grayscale conversion, (**l1**,**l2**) grayscale vertical flip, (**m1**,**m2**) grayscale horizontal flip, (**n1**,**n2**) grayscale center crop, (**o1**,**o2**) random brightness contrast, (**p1**,**p2**) random gamma, (**q1**,**q2**) hue saturation, (**r1**,**r2**) RGB shifting defines the random color change within red, green, and blue pixels, (**s1**,**s2**) random brightness, (**t1**,**t2**) random contrast, (**u1**,**u2**) motion blur, (**v1**,**v2**) median blur, (**w1**,**w2**) gaussian blur, (**x1**,**x2**) gaussian noise, (**y1**,**y2**) channel shuffling allows to rearrange these channels to create various visual effects, alter color balance, or apply artistic transformations to the image, and (**z1**,**z2**) coarse dropout which defines randomly setting rectangular black regions inside the images.

Kvasir-SEG contained 1000 images, of which 800 were randomly selected for training, 100 for validation, and 100 for testing sets. After pre-processing, this training set increased to 20,800 images. The CVC-ClinicDB dataset (https://polyp.grand-challenge.org/CVCClinicDB/ (accessed on 13 May 2023)) had 612 images, of which 489 were training, 61 were validation, and 62 were testing. After pre-processing, this training set increased to 12,714 images. All images were resized to 256 × 256 pixels to maintain consistency and reduce computational complexity. Data augmentation was applied only to the training set, while the validation set remained unchanged. The larger dataset led to decreased overfitting and enhanced generalization to previously unseen polyp images. All of the pre-process images are displayed in Figure 4.

### 3.3. IRv2-Net Architecture

**Figure 5:**
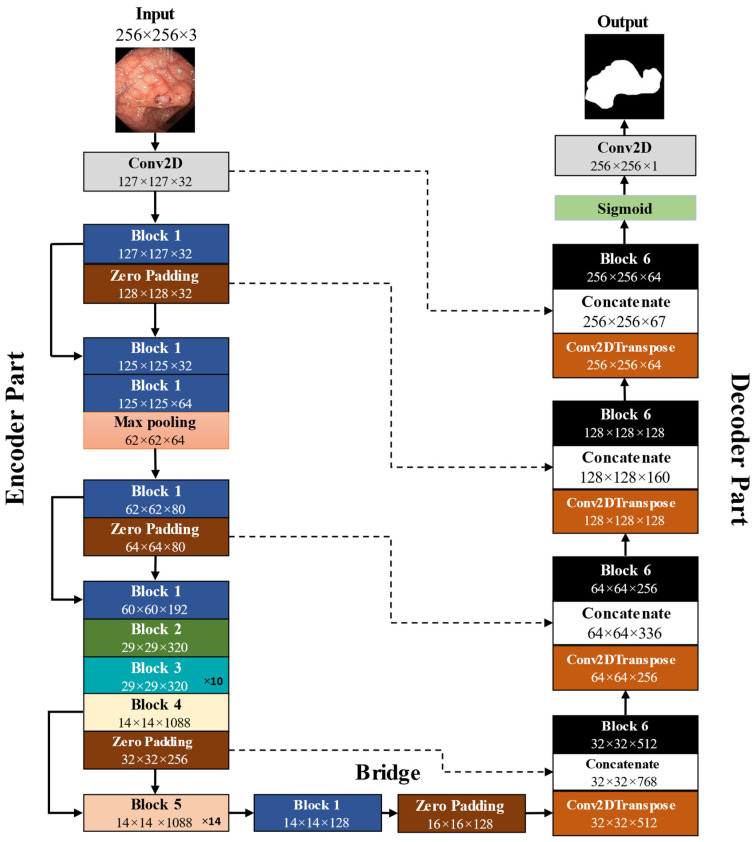
An overview of the architecture of IRv2-Net. The entire network consists of Encoder, Bridge and Decoder sections. Input Block is connected to a Conv2D Block followed by Block 1. Zero Padding Blocks are skip-connected to Concatenate Blocks. Block 1 to Block 6 are represented by different colors. Block 3 and Block 5 are repeating blocks. Block architectures are further explained in Figure 6.

The IRv2-Net architecture is a modification of the UNet and the Inception ResNet [39]. The Inception architecture and residual blocks (RBs) are combined to create Inception ResNet [40]. IRv2-Net incorporates the InceptionResNetV2 model, a robust convolutional architecture for image processing, with the UNet architecture. Figure 5 presents the illustration of the proposed IRv2-Net architecture.

In the top layer, the model takes an input image and performs convolutional and pooling operations to extract hierarchical features from the image. The input size of the images for the model is 256 × 256 × 3. The InceptionResNetV2 model is loaded with pre-trained weights from ImageNet. The UNet design comprises the encoder, bridge, and decoder components. The encoder component of a typical UNet model consists of four downsampling blocks that increase the number of filters while decreasing the spatial dimensions of the input image. Each block has two convolutional layers (CLs) and one max pool (MP) layer. For the CLs, the filter size is 3 × 3, and for the MPs, it is 2 × 2. MP is used to downsample the feature maps, reducing their width and height while increasing their depth. Inception-ResNet-v2 blocks replace the encoder blocks in the proposed architecture. After each Inception block, a filter expansion layer (1 × 1 convolution without activation) is added to adjust the depth of the filter to the input. These intermediate activation layers are extracted using the InceptionResNetV2 Keras model. The UNet design has four resolution levels, downsampling the input image from 256 × 256 to 32 × 32, and each convolution block (CB) has an increased or doubled number of filters (32, 64, 128, 256). The encoder uses CBs at various resolution levels to convert the input image into feature maps.

The encoder and decoder are connected by a bridge block built similarly to the downsampling. To generate the bridge, a specific activation layer from the InceptionResNetV2 model is extracted and zero-padded in the IRv2-Net architecture. The image size, produced by the bridge block that has 128 filters, is 16 × 16 × 128.

The architecture’s decoder blocks enlarge the image to recreate the segmentation map. This is achieved via transpose convolution (TC), concatenation, and CLs in four upsampling blocks. TC is used for upsampling, and upsampled feature maps and corresponding encoding layers are concatenated to create skip connections. This is followed by two convolution layers with Rectified-Linear-Unit (ReLU) activation. After each upsampling block, the feature map size increases as the number of filters drops, with filter sizes of 512, 256, 128, and 64 in the decoder branch. Until the input image resolution is found, these steps are repeated. 

The final convolution layer results in an image size of 256 × 256 × 64. The decoder branch takes an input image of 16 × 16 × 128. The segmentation map is obtained from the model’s output layer, which consists of a 1 × 1 convolutional layer followed by a sigmoid activation. The segmentation map assigns a class label to each pixel, representing the predicted class, and has the exact spatial dimensions (width and height) as the input image. At the final stage, an image size of 256 × 256 × 1 generates the binary segmentation mask.

### 3.4. Different Blocks of IRv2-Net Architecture

A detailed representation of the Inception ResNet architecture is necessary to analyze the IRv2-Net architecture thoroughly. The Inception ResNet, which combines Inception blocks and RBs, serves as the foundation for the architecture [40]. These blocks incorporate CLs, MPs, and other operations. Like the original UNet architecture, the proposed IRv2-Net retains a similar number of blocks while incorporating extra blocks from InceptionResNetV2 into the encoder portion. The decoder generates the final output by concatenating the convolutional results from the encoder into four parts while maintaining the original structure. There are 47 blocks altogether, of which 34 are for the encoder-bridge part and 13 for the decoder section. This produces four functional encoder units and four functional decoder units. Figure 5 provides information on each block’s input/output and feature output sizes. 

**Figure 6:**
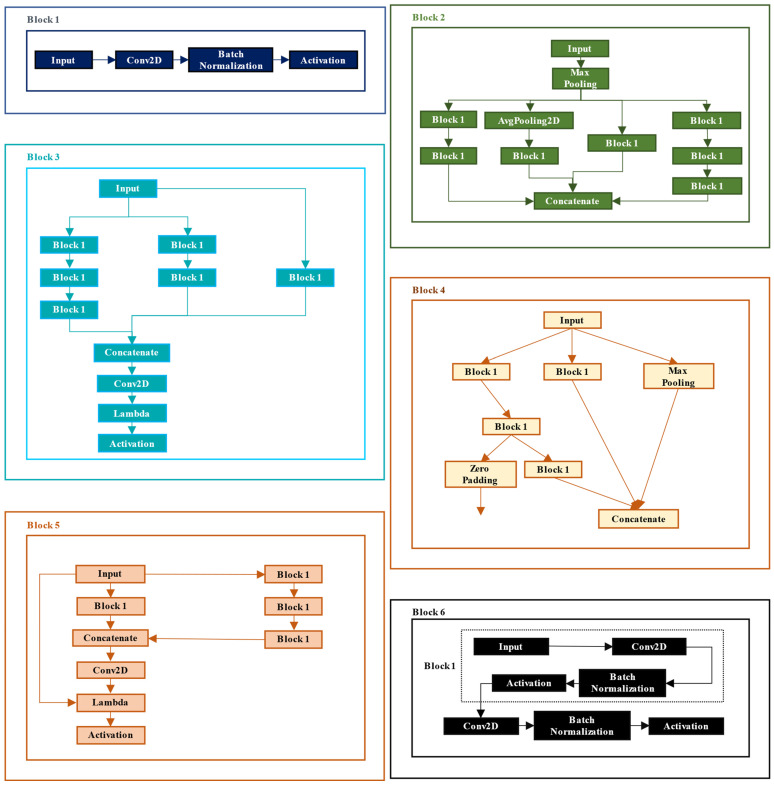
A detailed breakdown of each Block that is used in the IRv2-Net architecture.

Each block takes into account several concurrent convolutions with various kernel sizes, and each block’s outputs are combined to create a single layer of features. These parallel convolutions are contained within the sub-blocks shown in Figure 6. The performance of Inception ResNet is enhanced by balancing the filters at each stage. Inception blocks increase the network’s depth, improving the network’s capacity to learn intricate representations of image-to-mask mapping. At the same time, RBs address the problem of vanishing gradients and shorten model training time. Scaling of residuals and residual inception blocks (RIB) is included in the InceptionResNetV2 model as crucial components of its architecture. The InceptionResNetV2 model, offered by the Keras package, contains these preconfigured blocks. Particularly, in deep architectures with several layers, they are essential in preventing gradient vanishing during backpropagation [41]. CLs, MPs, and ReLU activation functions make up the blocks. CLs extract features from images by sliding convolution kernels across them with specified stride parameters. 

Each Inception block in the architecture is followed by a residual connection, where the block’s output is added to its input to create the residual connection. This simplifies optimization and eliminates vanishing gradients while facilitating gradient flow during training. The filter bank’s dimensionality is also adjusted to match the input depth after each Inception block by a filter expansion layer, a 1 × 1 CL without activation, assuring compatibility with the subsequent layers. The inclusion of residual connections and scaling of residuals in deep architectures is particularly beneficial for avoiding gradient vanishing during backpropagation, especially in models with many layers [41].

The decoder part comprises blocks of convolution, TC, and concatenation. To achieve the same size for concatenating features, zero padding is used. The Input Layer, which inputs an image with a dimension of 256 × 256 × 3, is the first component of the encoder. Then, Block 1 of the encoder portion is iterated through an MP three times. The First Encoder Block is Block 1, with a feature output size of 127 × 127 × 32. The Conv2D, Batch Normalization (BN) and activation layers comprise Block 1’s structure (see Figure 6). Block 1’s 127 × 127 × 32 feature size is also zero-padded, resized to 128 × 128 × 32, and skip-connected to a Concatenate layer of a decoder block. Block 1 is repeated twice after the MP, then Blocks 2 and 3 are repeated only once. Block 1 is zero-padded as before and resized to 64 × 64 × 80 before being skip-connected to a decoder block. Block 1’s feature output size was 62 × 62 × 80. The second encoder block is this one. Then, Block 3 is repeated an additional ten times. The feature output size is 29 × 29 × 320, which is the input of Block 4 as the fourth encoder part. Block 4 moves on to Block 5 after providing two outputs, one for concatenation and the other for zero-padding, to enlarge the feature output to 32 × 32 × 256 and skip-connect it with another decoder block. A regular InceptionResNetV2 has 20× Block 5, but we used 14× and obtained superior results. After 14 repetitions, Block 5’s feature output size is 14 × 14 × 1088, which feeds into the bridge block. As the bridge block, the following Block 1, with feature output size 14 × 14 × 128, receives zero padding from providing an output size of 16 × 16 × 128. The Conv2DTranspose layer of the first Decoder Block is then attached to this block. The crucial part of the network’s output, Block 6, creates the final mask image by concatenating the input and output of the UNet encoder–decoder block. Block 6 contains all the layers from Block 1 in its structure and more layers.

The details of Blocks 1 to 6 are displayed in Figure 6, while the other blocks have Block 1 in their structure. The decoder branch of the IRv2-Net architecture contains TC, concatenation, and CLs, forming a total of 13 segments (see Figure 5), thus producing four functional decoder parts and one output layer. Combining a Conv2DTranspose layer, a Concatenate layer, and Block 6 produces a decoder block unit. In order to obtain a total of four functioning decoder blocks, each unit is repeated four times. The initial Conv2DTranspose layer is used in this section to upsample the zero-padded feature map from the bridge block directly. The following four Concatenate layers skip-connect four upsampled Conv2DTranspose feature maps with corresponding zero-padded feature maps from encoder layers at the same resolution level. This process gradually recovers the spatial resolution of the feature maps.

The first Decoder block incorporates skip connections with zero-padded Block 6 having an output size of 16 × 16 × 128 by performing concatenation of the upsampled feature map (32 × 32 × 512) with feature map derived from the corresponding encoding layers (Block 4) at the same resolution level (32 × 32), but with a different filter size (256). Then, comes Block 6, which has two convolution layers, two batch normalization layers, and two activation layers. When the number of filters is reduced by half, the image size increases by two times. By the fourth Decoder block, the number of filters in the decoder branch drops from 512 to 256 to 128 to 64. These operations are repeated until the 256 × 256 × 64 feature output is reached. The second, third, and fourth Decoder blocks each have a feature resolution of 64 × 64 × 256, 128 × 128 × 128, and 256 × 256 × 64, respectively. Only a Conv2D layer with sigmoid activation is present in the fifth layer, producing a segmentation mask with 256 × 256 × 1. This is the predicted output mask.

### 3.5. Test time Augmentation (TTA) Technique

**Figure 7:**
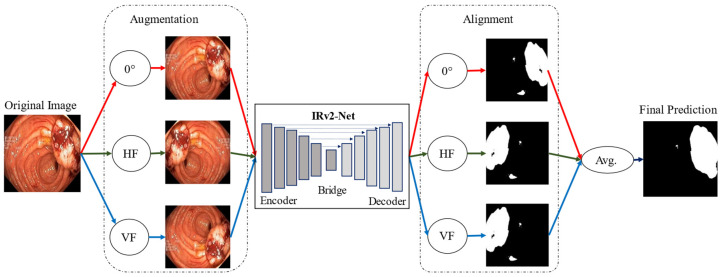
An illustration of the architecture for the proposed Test Time Augmentation where red arrow for the original sample, HF-horizontal flip (green arrow) and VF-vertical flip (blue arrow) are presented.

In machine learning, test time augmentation (TTA) is a technique that is frequently used to enhance the performance of models during the testing or inference phase, particularly in computer vision problems. TTA entails applying techniques to augment the test data before averaging the sample predictions with the newly added data to produce the final prediction [42]. Because it generates predictions that are more precise and consistent, the test time extension is significant. TTA helps to fix problems like overfitting by adding more variety to the reasoning process. Regarding polyp separation, TTA can be especially helpful. Finding and identifying polyps, i.e., abnormal growths, in medical imaging like colonoscopy images is the goal of polyp segmentation. The model can consider potential variations in how polyps appear, such as how they are positioned or where they are in the image, by employing TTA during the testing phase. By doing this, the accuracy and reliability of the segmentation findings are maintained. The most successful TTA technique, according to the tests performed in this study, is flipping. In Figure 7, a TTA illustration is displayed. The model predicts each image’s final orientation for rotations of 0 degrees, horizontal flip, and vertical flip, respectively. After lining up, the mean value of all augmentation is calculated. Algorithm 1 represents the overall TTA approach with the IRv2-Net model.
**Algorithm 1:** Proposed computational TTA approach on the test sample images.**Input:** The Pre-trained Model is defined as ‘*M*’ on in-distribution data. The augmented terminology is denoted as ‘*A*’. The test samples set of images data $X_{1},\;X_{2\;}{,X}_{3},\;\ldots \;{,X}_{n}$; which contains in-distribution data and out-distribution data combinedly. **Output:** Predicted samples with distributed mean score as              $S{(X}_{1}$), $S{(X}_{2}$), $S{(X}_{3}$), …, $S{(X}_{n}$); 1: **Begin** 2: **For loop** is executed: *i* = 1 to *n*
**do**
3:    Data augmentation for each input sample set, $X_{i}$= $\{X_{1},\;X_{2\;}{,X}_{3},\;\ldots \;{,X}_{n}$}; 4:    Converted to horizontal flip (HF) with *M* to obtain ${A(X}_{i}),$ respectively. Similarly, vertical flip (VF) is generated for each sample to ${A(X}_{i})$; 5:    Feed $X_{i}$ to ${A(X}_{i})$ into model *M*, continuously calculate ${M(X}_{i}),\;\;{M(A(X}_{i}))$ through forward passes; 6:    Mean result, $S(X_{i})=\frac{\sum_{i=1}^{n}{M(A(X}_{i}))\;}{n}$; 7: **end for** 8: **End**

The notation “$X_{i}$= $\{X_{1},\;X_{2\;}{,X}_{3},\;\ldots \;{,X}_{n}$}” signifies a dataset with multiple samples, where $X_{i}$ is each sample from the original dataset, incorporating operations like horizontal flip and vertical flip. The algorithm iterates over *i*, where each *i* corresponds to a different data instance. The notation “$A(X_{i})$” represents the application of image augmentation to original sample $X_{i}$. “Feed $X_{i}$ to $A(X_{i})$ into model *M*” denotes passing each original image $X_{i}$ through both $A(X_{i})$ and model *M* for predictions. The updated “$\;S\left(X_{i}\right)$” notation clarifies that *i* refers to various test data, specifically HF, VF, and original, executed via model M using TTA. The purpose of this mean calculation is to aggregate predictions from multiple augmented inputs for TTA.

## 4. Experimental Analysis

This part demonstrates the evaluation metrics, a loss function, and a detailed overview of the experimental setup and configuration.

### 4.1. Evaluation Metrics

A thorough analysis of a relevant study guided our selection of evaluation metrics, including Dice Coefficient (DSC), Intersection Over Union (IoU) or Jaccard coefficient, precision, recall, and accuracy. To assess the performance of the proposed model, these metrics were applied to both datasets. The formula for these metrics is provided in detail here. [43]. Within the formulation of each metric, the abbreviations TP, FP, TN, and FN correspond to true positive, false positive, true negative, and false negative, respectively.

(1) $$DSC=\frac{2\times TP}{2\times TP+FP+FN}$$

(2) $$IoU=\frac{TP}{TP+FP+FN}$$

(3) $$Recall=\frac{TP}{TP+FN}$$

(4) $$Precision=\frac{TP}{TP+FP}$$

(5) $$Accuracy=\frac{TP+TN}{TP+TN+FP+FN}$$

### 4.2. Binary Cross Entropy Loss

The loss functions are necessary for segmentation models because they direct the optimization process and produce accurate and exact segmentation outcomes. For binary segmentation tasks, the loss function known as log loss or binary cross entropy loss (BCE_loss_) is frequently utilized [44]. BCE_loss_ can be calculated using the formula:

(6) $$BCE_{loss}=-(y\times log\left(p\right)+(1-y)\times log(1-p))$$

Here, *p* stands for the anticipated probability of the positive class, and *y* stands for the ground truth label (0 or 1). The binary cross entropy loss incentivizes the model to provide right predictions with a higher probability while penalizing wrong ones.

### 4.3. Experimental Setup and Configuration

The same settings are used for training all models (UNet, Attention-UNet, ResUNet, ResUNet++, and IRv2-Net). The project source code available: https://github.com/Faysal425/IRv2-Net (accessed on 11 August 2023). The essentials of device configuration are summarized in Table 1.

The Nadam optimizer adjusts the learning rate based on gradient moments and utilizes nesterov momentum to improve convergence and handle sparse gradients [45]. Table 2 presents the hyperparameters in detail.

## 5. Experimental Results and Discussion

After the experiment, the results were analyzed quantitatively and qualitatively. The proposed model was compared with other models, including UNet, Attention-UNet, ResUNet, and ResUNet++, after a successful training phase. It was difficult to compare the outcomes directly because different researchers utilized distinct testing samples. Still, IRv2-Net evaluation time on test dataset (without TTA) is around 50 s, which is comparably low (500–1000 ms per sample).

### 5.1. Kvasir-SEG Dataset

**Figure 8:**
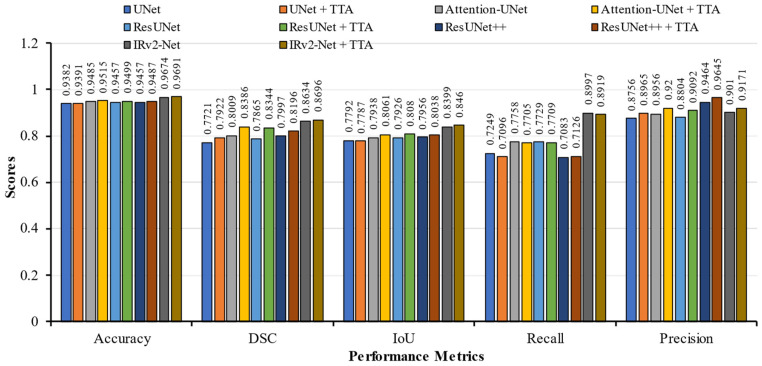
An illustration of performance metrics including accuracy, DSC, IoU, recall, and precision on Kvasir-SEG trained models.

Table 3 displays the evaluation results. In this instance, all models were trained using the Kvasir-SEG training set. By incorporating the TTA postprocessing approach, the detection rate of the proposed IRv2-Net architecture was enhanced. In IRv2-Net + TTA, the accuracy, DSC, IoU, recall, and precision scores were 96.91%, 86.96%, 84.60%, 89.19%, and 91.71%, respectively. In contrast to the post-processing TTA method, the base IRv2-Net model achieved a slightly higher recall score of 89.97%. Notably, the IRv2-Net + TTA model outperformed the TransNetR SOTA model by 5.24% points in IoU and 0.84% in the recall. Similarly, this model outperformed the other SOTA DDANet models in terms of DSC, IoU, recall, and precision score by 1.37%, 7.80%, 0.44%, and 5.76%, respectively. Also, another SOTA FCN + CNN model performed better in DSC, but at IoU, our base model and TTA model achieved the highest score, approximately 5.12%. Regarding precision, the Resunet++ model scored 94.64%, while the IRv2-Net + TTA model scored 91.71% (2nd highest). The accuracy and DSC scores of the Attention-UNet and ResUNet++ models in the same test sets were lower. With TTA, the accuracy result for IRv2-Net increased to 96.91% from 96.74%. On the Kvasir-SEG dataset, the proposed model produced significantly superior segmentation results than other models. Furthermore, other TTA-integrated model performance is also lower than the IRv2-Net + TTA model. Figure 8 depicts a bar chart of the Kvasir-SEG test set performance metrics.

### 5.2. CVC-ClinicDB Dataset

Table 4 presents a comprehensive evaluation of diverse segmentation models with a specific emphasis on elucidating the efficacy of the proposed models. In the CVC-ClinicDB dataset, where models predict distinct regions within images, a range of performance metrics, including accuracy, DSC, IoU, recall, and precision, are employed to measure the model proficiency. The proposed IRv2-Net architecture had an improved detection rate by using the TTA post-processing approach, where the accuracy, DSC, IoU, recall, and precision scores were 98.55%, 93.75%, 89.49%, 91.51%, and 94.82%, respectively. Remarkably, with TTA, 0.4% higher DSC scores were achieved from the Irv2-Net model. This model’s DSC score of 93.75% surpassed HarDNet-MSEG, TransUNet, Double Attention ResUNet, CaraNet, and Dilated ResFCN models. At TransUNet, the second-highest DSC was achieved with a noticeable improvement of 0.26% compared to Irv2-Net + TTA. However, the Irv2-Net model showed a remarkable improvement of 0.53% in IoU scores, while the Irv2-Net + TTA model had a 0.88% improvement. Similarly, the Irv2-Net model outperformed the CaraNet model by 0.53% in the IoU scores, which improved to 0.88% with TTA. The precision and recall scores were significantly improved in the proposed IRv2-Net model compared to the Dilated ResFCN by achieving 11.35% and 14.21% (without TTA), respectively. This improvement was even more with TTA, which simultaneously showed 11.49% and 14.57% improvements in precision and recall. The recall and precision scores also demonstrated a competitive improvement of approximately 10.89% and 13.87% from UNet with TTA and 16.71% and 0.99% from ResUNet++ with TTA.

**Figure 9:**
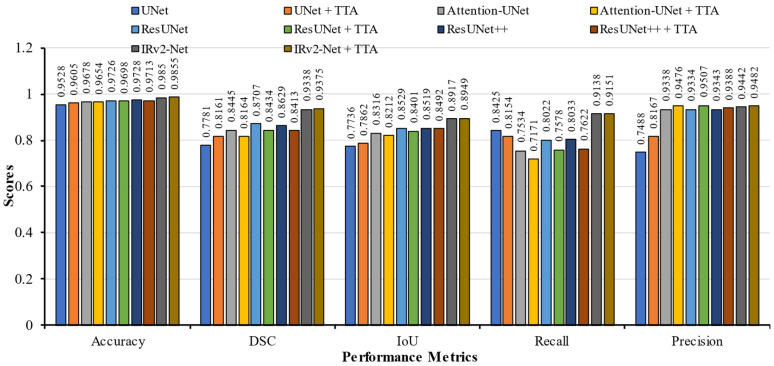
An illustration of performance metrics including accuracy, DSC, IoU, recall, and precision on CVC-ClinicDB trained models.

Notably, our IRv2-Net with TTA achieved the highest performance metrics scores than other TTA-integrated models, which is highly significant for polyp detection. Based on this analysis, the proposed model showed the highest quantitative performance on the CVC-ClinicDB test set. Figure 9 displays a bar graph of the performance metrics.

The differences in performance between datasets (as seen in Table 3 and Table 4) stem from the unique characteristics of each dataset. The Kvasir-SEG dataset is more complex due to smaller, irregular polyps with diverse appearances. This complexity challenges accurate segmentation. Additionally, the Kvasir-SEG dataset lacks alignment, hindering the model’s ability to capture polyp intricacies. Conversely, the CVC-ClinicDB dataset benefits from better alignment and consistent image quality, enhancing segmentation accuracy. The distinct image formats (colored PNG for Kvasir and TIFF for CVC-ClinicDB) are also a reason for performance variation.

### 5.3. Cross Dataset Performance Evaluation

Cross-dataset evaluation is crucial for assessing the robustness and generalizability of a trained model. The diversity of datasets enables evaluation of how well a model interacts with the real world and aids in identifying and correcting biases in the training dataset. The best model can be found through benchmarking and contrasting various models.

#### 5.3.1. Model Trained on Kvasir-SEG and Tested on CVC-ClinicDB Dataset

Firstly, The CVC-ClinicDB samples, consisting of 62 original samples and the same number of mask images, were first used to evaluate the Kvasir-SEG-trained models. Figure 3 displayed a dataset preview, illustrating the vast differences in image quality, polyp morphologies, and behavioral conditions. If the proposed model can precisely segment the polyp area under a wide range of worst conditions, it is superior to existing models. Table 5 displays the quantitative outcomes of multiple models evaluated against the CVC-ClinicDB dataset. Notably, the UNet model yielded the worst results: 30.04% DSC and 53.89% IoU. Attention-UNet received 48.18% DSC and 63.1% IoU, respectively. The proposed IRv2-Net + TTA obtained the highest scores for DSC (54.69%), IoU (64.57%), and recall (66.34%).

#### 5.3.2. Model Trained on CVC-ClinicDB and Tested on Kvasir-SEG Dataset

Secondly, the model’s performance trained on the CVC-ClinicDB was evaluated using Kvasir-SEG samples. Table 6 displays the quantitative results of the models tested on the Kvasir-SEG dataset. The IRv2-Net and IRv2-Net + TTA models had the highest accuracy (93.69% and 93.55%, respectively). The DSC score was where the base model exceeded the TTA approach, outperforming it by 1.84% and raising the IoU scores by 0.53%. The highest recall score, 69.66%, was obtained at IRv2-Net. The TTA technique resulted in a maximum precision score of 91.12%.

### 5.4. Qualitative Analysis

On the test set predictions, the Kvasir-SEG and CVC-ClinicDB datasets were evaluated. To gain a complete understanding, we divided the dataset samples into two separate groups. The highest and lowest 16 samples were chosen based on their DSC scores. The red frames are the selected images for prediction. These selection criteria were implemented based on the size of the polyps. From the input samples, the UNet, Attention-UNet, ResUNet, ResUNet++, IRv2-Net, and IRv2-Net + TTA models were used to generate the mask.

#### 5.4.1. Kvasir-SEG Dataset

**Figure 10:**
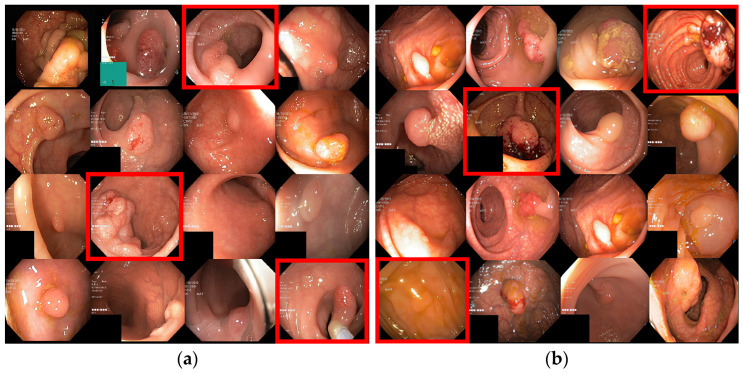
This figure depicts the Kvasir-SEG test samples, which include (**a**) the samples with the highest DSC scores, and (**b**) the samples with the lowest DSC scores. Red-boxed images above are considered as the samples used to generate model predictions.

The Kvasir-SEG test samples, including three samples from each set, were used to create predictions (see Figure 10a,b). The highest DSC samples are presented in Figure 10a, whereas the lowest DSC samples are shown in Figure 10b. All models have trouble recognizing polyps in lower DSC samples.

**Figure 11:**
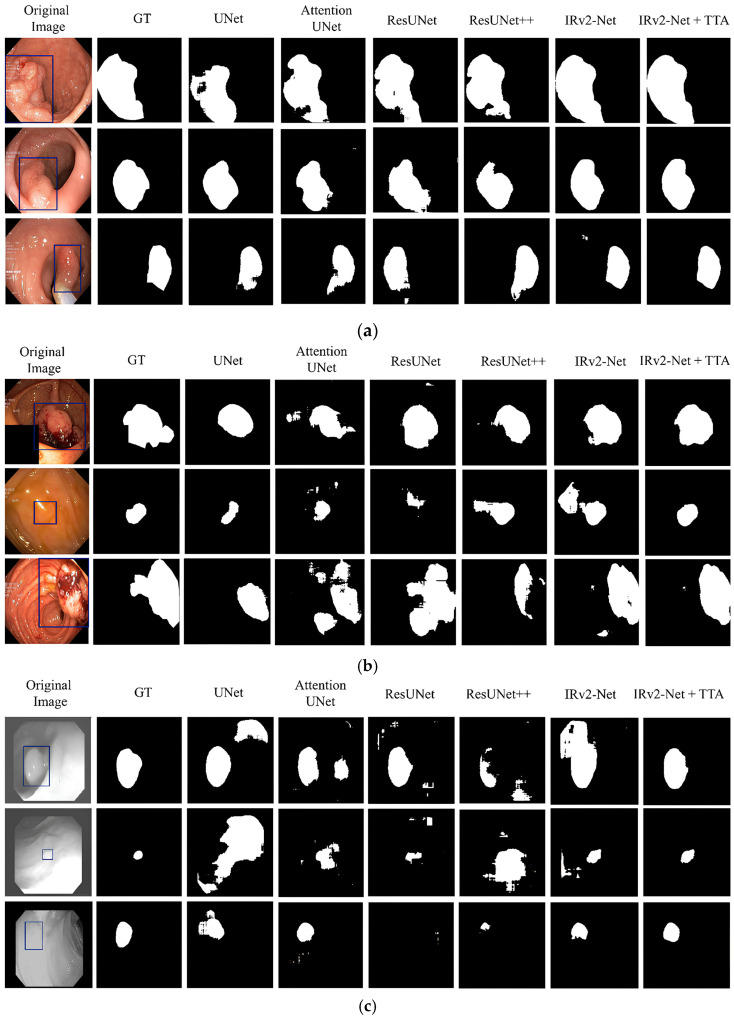
An illustration of predicted masks, where (**a**) prediction on top scored images (larger, medium, and small polyps), (**b**) prediction on bottom scored images (medium, flat and larger polyps), and (**c**) prediction on the CVC-ClinicDB dataset (medium, flat and small polyps). Blue-boxed regions signify the polyp regions in the Ground Truth mask.

- Top DSC samples: Figure 11a displays the selected top DSC samples to generate predictions along with the appropriate ground truth (GT), where the blue box on the original image designates the original polyp region according to the GT mask. The segmentation area was then produced again using several models. The maximal polyp area was appropriately detected from the input using the IRv2-Net + TTA model. The IRv2-Net (*w*/*o* TTA) model can also detect the polyp region, but the TTA method increased the accuracy by enhancing the masks and averaging the results.
- Lowest DSC samples: The lower DSC samples’ predictions are presented similarly in Figure 11b. The suggested model, however, outperformed others in terms of performance in identifying the smallest polyp regions. The most considerable foreground in the predictions was identified by the IRv2-Net model, and it was more precisely developed using TTA. As shown in Figure 11b (2nd row), the IRv2-Net with TTA successfully segmented flat polyp areas, but the maximum model failed to generate an accurate prediction. For others, larger polyps (first and third samples) were easily identifiable by the proposed model.
- Cross testing: For model generalizability, we tested the CVC-ClinicDB test samples on our system. Remarkably, these Kvasir-SEG-trained models showed satisfactory prediction results compared to others on these data. The projected performances, based on unseen data, are shown in Figure 11c. The IRv2-Net + TTA model predicted entirely unseen data more accurately than others. In conclusion, our proposed model performed much better qualitatively for comparison and cross-prediction.

#### 5.4.2. CVC-ClinicDB Dataset

**Figure 12:**
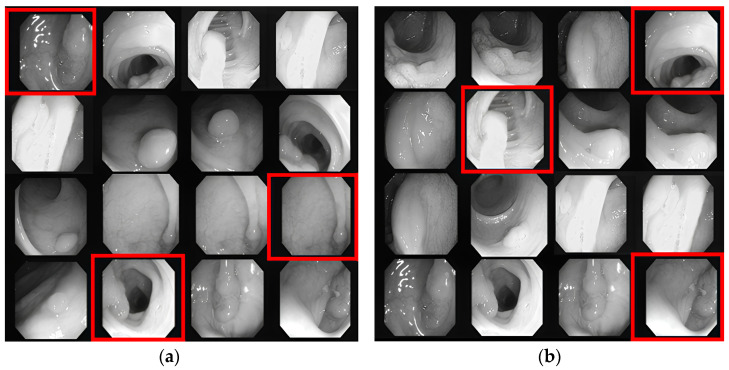
This figure depicts the CVC-ClinicDB test samples, which include (**a**) the samples with the highest DSC scores, and (**b**) the samples with the lowest DSC scores. Red-boxed images above are considered as the samples used to generate model predictions.

The proposed model was trained on the CVC-ClinicDB dataset and tested on a test set from the same dataset. The selection criteria for the sample were based on the presence of large, saturated, and flat polyps. Figure 12a displays the highest 16 DSC samples, whereas Figure 12b displays the lower DSC samples. The results showed that all models can capture larger polyps but faced difficulty capturing regions of oversaturated and flat polyps.

**Figure 13:**
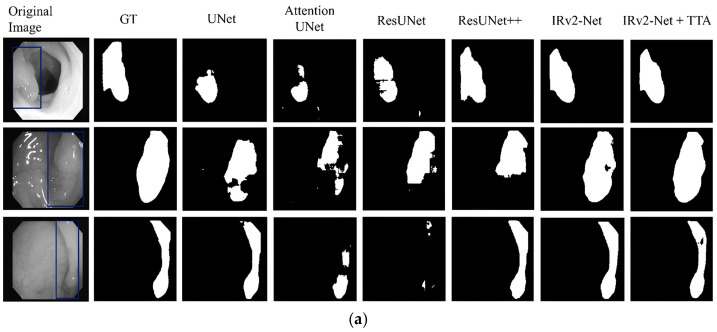

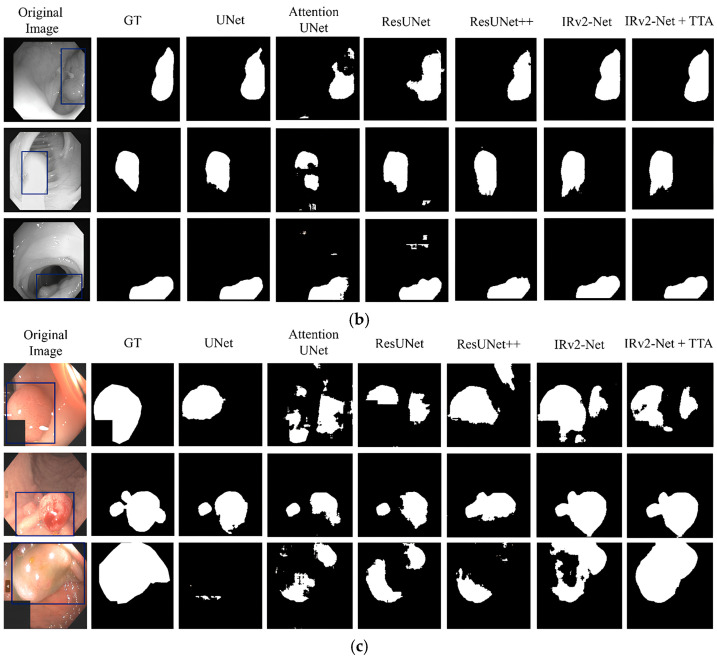
An illustration of predicted polyp masks (blue boxes define the ground truth polyp areas), where (**a**) prediction on top scored images (medium, larger and flat polyps), (**b**) prediction on bottom scored images (medium, oversaturated and flat polyps), and (**c**) prediction on the Kvasir-SEG dataset (medium, small and larger polyps).

- Top DSC samples: The ground mask sections of the polyp area are shown in blue boxes in Figure 13a, which shows the prediction of the selected top three samples. In oversaturated data (first row), most models failed to correctly identify the precise polyp area because of high contrast on the image. The IRv2-Net and TTA models correctly identified the mask, whereas others failed.
- Lowest DSC samples: The selected samples of lower DSC scores are shown in Figure 13b. The polyp masks created by the IRv2-Net + TTA model were superior to those created by other models. Remarkably, saturated (second row) and flat (third row) type polyps were accurately detected in the proposed model. Utilizing TTA, these predicted regions are more precisely reshaped by deleting excessive areas.
- Cross testing: The Kvasir-SEG dataset was tested to observe the model’s qualitative behavior. Then, Kvasir-SEG test samples were used to put these trained models to generate predicted masks. The expected outcomes based on unobserved data are shown in Figure 13c. For predicting unseen data, the IRv2-Net + TTA model showed better performance. Overall, our suggested approach considerably enhanced the quality of results for both datasets and cross-prediction.

### 5.5. ROC-AUC Curves

An essential evaluation parameter for polyp segmentation is the ROC-AUC, which measures how well the model can discriminate between polyp and non-polyp regions. The ROC curve supports improved threshold optimization and polyp segmentation optimization. The ROC-AUC metric is crucial in summarizing a model’s performance, aiding in effective decision-making. It has been estimated for cross-dataset, CVC-ClinicDB, and Kvasir-SEG evaluations.

**Figure 14:**
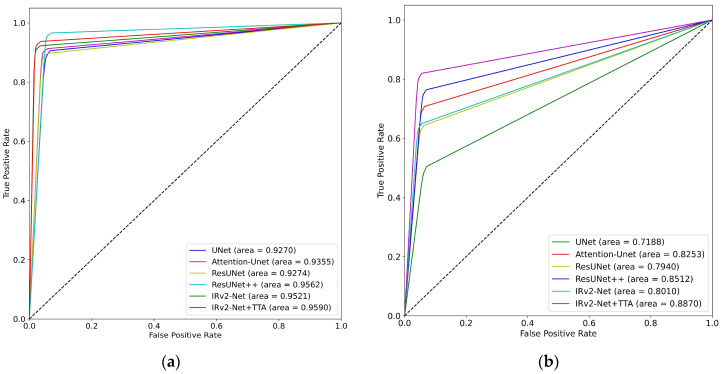
ROC-AUC curves include (**a**) trained and tested on Kvasir-SEG dataset, and (**b**) trained on Kvasir-SEG and tested on CVC-ClinicDB dataset.

**Figure 15:**
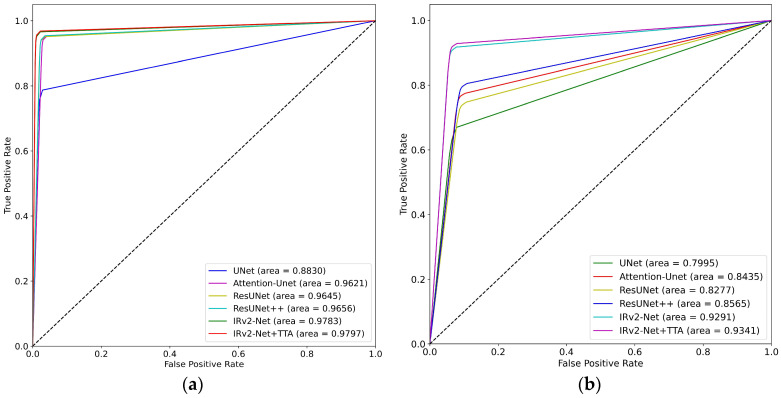
ROC-AUC curves include (**a**) trained and tested on CVC-ClinicDB dataset, and (**b**) trained on CVC-ClinicDB and tested on Kvasir-SEG dataset.

The results of the Kvasir-SEG trained model are shown in Figure 14a, where the IRv2-Net + TTA combination attained a maximum area score of 0.9590. Additionally, Figure 14b displays the highest score (0.8870) on cross-testing data. Figure 15 shows that the IRv2-Net + TTA model outperformed all other models with an exceptional score of 0.9797 on the CVC-ClinicDB dataset following its training and testing. On previously unseen Kvasir-SEG data, the highest (0.9341) score was obtained. The evaluation demonstrates that both similar-type and unknown data benefit from the performance of the IRv2-Net + TTA model.

### 5.6. AUC-PR Curves

Precision quantifies the precision of positive predictions made by a model, while recall gauges its proficiency in recognizing all positive occurrences. Demonstrating the precision (y-axis) and recall (x-axis) of a model for image segmentation, the AUC-PR curve offers a visual representation [60]. Accurate polyp segmentation in endoscopic images relies on striking a balance between accuracy and recall, as seen by the AUC-PR curve. A model makes the most correct predictions with high precision and recall, but the opposite may be evident for a model with poor precision and high recall [61]. High precision and recall in polyp segmentation indicate accurately identifying a restricted number of polyp regions while minimizing false positives. In contrast, a model with low precision and high recall may incorrectly identify a more significant number of polyp regions. A perfect polyp segmentation model accomplishes high precision and recall, allowing for the precise detection of polyp regions.

**Figure 16:**
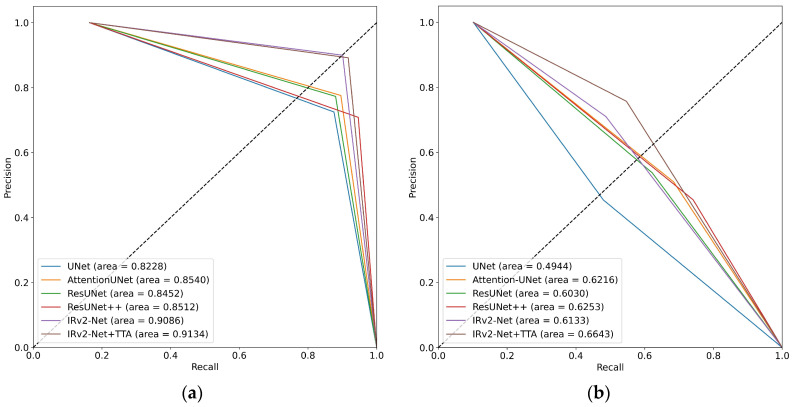
AUC-PR curves including (**a**) trained and tested on the Kvasir-SEG dataset, and (**b**) trained on Kvasir-SEG and tested on the CVC-ClinicDB dataset.

**Figure 17:**
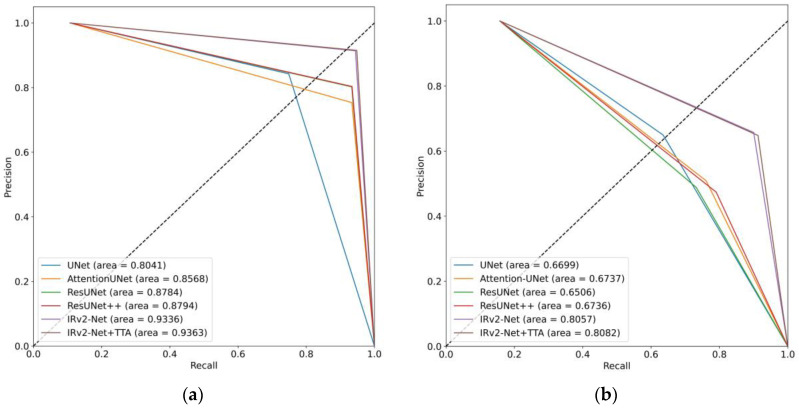
AUC-PR curves including (**a**) trained and tested on the CVC-ClinicDB dataset, and (**b**) trained on CVC-ClinicDB and tested on the Kvasir-SEG dataset.

Figure 16 shows that the AUC-PR values on the Kvasir-SEG dataset ranged from 0.8228 to 0.9134. IRv2-UNet + TTA was the model with the highest AUC-PR score, scoring 0.9134. This demonstrates improved performance in terms of both precision and recall. It obtained the highest score (0.6643) on the CVC-ClinicDB dataset. Figure 17 shows that the AUC-PR values on the CVC-ClinicDB dataset ranged between 0.8041 and 0.93. IRv2-UNet + TTA obtained the highest AUC-PR score of 0.9363, indicating its superior precision and recall performance on this dataset. This model obtained the maximum score (0.8082) on the Kvasir-SEG data set. In both instances, the IRv2-Net + TTA model obtained the highest area coverage compared to others.

### 5.7. Graphical User Interface (GUI)

**Figure 18:**
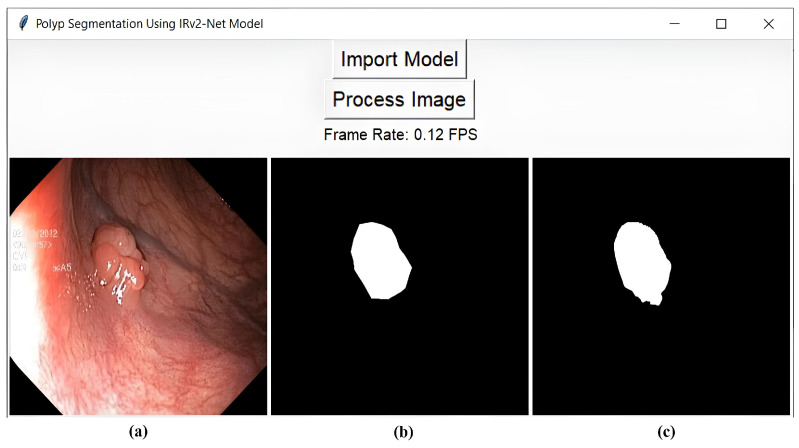
Visualization of GUI interface includes (**a**) the original sample, (**b**) the ground truth, and (**c**) the predicted mask.

The graphical user interface enables users to interact with the segmentation system in real-time tests, allowing them to select samples from various patients and readily visualize segmentation results. This user-friendly interface enhances the client experience and reduces the need for endoscopists to possess technical expertise or programming skills. Based on this visual feedback, adjustment is possible by displaying the original colon images, ground truths, predicted masks, and other variants of model weight. This graphical representation enables endoscopists to evaluate the segmentation’s accuracy and make precise decisions. In our research, we developed a graphical user interface (GUI) framework that displays segmentation results based on selecting various model weights without the need for programming intervention. Algorithm 2 briefly describes the GUI’s functioning steps. Figure 18 depicts the framework’s interface with the test samples and FPS counter. The developed GUI tool operates on a frame-level prediction basis, with each image taking approximately 500 to 1000 ms response time from the GUI. The tool does not require a GPU or any other accelerator to function.
**Algorithm 2:** Functioning steps for the proposed interface.1: **Begin** 2: Initializing the global variable: ‘M’, and ‘P’ represented model and path. 3: **Function I():**      // importing model weight .h5 4:    If P is not empty: 5:      M = Load(P) 6:      Display(“Model imported successfully!”) 7: **Function P():**      // processing the image and generating the mask 8:    If M is null: 9:      Display(“Please import a model first!”) 10:    Return 11:    I = Dialog(“Select Image File”) 12:    If I is not empty: 13:      Img = Load(I) 14:      start_time() 15:      ImgArr = Preprocess(Img) // performing image resizing to 256 × 256 16:    PMask = Postprocess(Mask)  // post-processed mask 17:      end_time() 18:    GTMask = Load(GT) 19:    processing_time = end_time–start_time 20:    frame_rate = 1.0/processing_time 21:   ClearWindow() 22: **Main():**
23:   Create GUI window 24:   Add “Import Model” with I() as the callback 25:   Add “Process Image” with P() as the callback 26:   Start GUI event loop 27: **Exit**

The following algorithm provides the user with a graphical user interface (GUI), which allows them to import a model weight, process an image, generate segmentation masks, and visualize the results, all while tracking processing times and frame rates. The algorithm starts by initializing two global variables, ‘M’ for the model and ‘P’ for the model’s path. The ‘I’ function imports the pre-trained model weights in the ‘.h5’ format. It checks whether the model path ‘P’ is empty, then loads the model ‘M’ and displays a success message. The ‘P’ function handles image processing and mask generation. If the model ‘M’ is null (indicating that no model has been imported), it displays a message to import a model first. Suppose an image file is selected through a dialog box, in that case, it loads the image, preprocesses it by resizing it to 256 × 256 pixels, and generates a post-processed segmented mask using the loaded model. The processing time for this operation is measured using a start and end time calculation. The ground truth mask is also loaded for comparison. The GUI window is cleared, and the ‘Main’ function sets up the GUI window, adds options to import the model and process an image, and starts the GUI event loop to enable user interactions. The algorithm concludes by exiting the process.

### 5.8. Challenges & Limitation

Several issues can arise when segmenting polyps, such as problems preparing the colonoscopy environment, camera collection criteria, too much noisy information, and variable angle morphology. These characteristics have a significant impact on the model’s performance. Endoscopists continue to have difficulty pinpointing a polyp’s precise location from an image. The proposed IRv2-Net model, enhanced with test time optimization, effectively identifies the regions of the original samples with the maximum polyp concentration. This model can aid endoscopists in two significant ways: by automating the detection of lesions and reducing the need for radiologists. The model outperforms others in detecting oversaturated and flat or sessile polyps, as demonstrated by quantitative, qualitative, ROC-AUC, and AUC-PR curve analyses. The proposed model can be implemented at the clinical level due to its substantial benefits.

There is a limitation to this effort, however. All images were initially resized using a downsampling operation, reducing their resolution from (332 × 487–1920 × 1072, 288 × 384) to 256 × 256 pixels. This resizing procedure may prevent the accurate identification of numerous small lesions. Downsampling entails a reduction in image dimensions, which can lead to the loss of intricate details and subtle contrasts within the Region of Interest (ROI). Due to their inherently complex nature, small lesions may be affected by this process. The downsampling procedure may lead to the blurring or merging of minute structures, making it challenging for automated systems or even human observers to discern these smaller lesions within the images precisely. Planned future work will involve the application of a crop function to tiny regions as the input for the model. This methodology will facilitate the extraction of accurate contextual and specific insights.

## 6. Conclusions

This paper discusses the IRv2-Net architecture for polyp segmentation in colonoscopy. Utilizing the TTA interface improved segmentation performance. The IRv2-Net model combined the UNet model and the retrained InceptionResNetv2 model. It consisted of four interconnected encoder blocks, a bridge, and four decoder blocks. The segmented lesion was obtained using TTA by averaging the results of the previous phases. The Kvasir-SEG, CVC-ClinicDB, and cross datasets were evaluated and compared to SOTA models such as UNet, Attention-UNet, ResUNet, ResUNet++, and others. In quantitative, qualitative, ROC-AUC, and AUC-PR evaluations, the IRv2-Net + TTA model performed exceptionally well.

Moreover, our GUI application was successfully used throughout the colonoscopy exam to increase accuracy. The most significant advantage of the proposed method is its ability to precisely identify microscopic, oversaturated, flat, or sessile polyps, which are frequently overlooked during colonoscopy examinations. The IRv2-Net + TTA method is beneficial for clinical studies that require real-time data collection but do not necessitate extensive research. Before relying on the model, it is essential to understand its limitations in terms of applicability, as it may yield subpar results in certain situations. In order to establish the system’s utility, our CAD technique will be evaluated clinically in the future, following the elimination of intestinal issues and field testing.

## Figures and Tables

**Table 1:** Hardware and Software setup for experimental analysis.

Name	Parameters
Programming Language	Python 3.9
Environment	Microsoft Visual Studio Code 1.78.2
Deep Learning Framework	TensorFlow 2.12.0, Keras 2.12.0
Processor	Intel Core i7-10700K @5.00 GHz
Installed RAM	32 GB
GPU	NVIDIA GeForce, RTX 2080 Ti 11 GB
Operating system	Windows 11 Pro

**Table 2:** Comparative outline of Hyperparameter Settings with proposed model where (-) denotes the null value.

Method	Trainable Parameter	Learning Rate	Momentum	Optimizer	Batch Size	Loss	Threshold
FCN8 [46]	134,270,278	1 × 10^−4^	-	SGD	8	Cross-entropy	-
HRNet [47]	9,524,036	1 × 10^−4^	-	Adam	8	Dice loss	-
PSPNet [48]	48,631,850	1 × 10^−2^	-	SGD	8	Cross-entropy	-
DeepLabv3+ [49]	ResNet50: 39,756,962	1 × 10^−2^	-	SGD	8	Cross-entropy	-
	ResNet101: 58,749,090	1 × 10^−3^	-	SGD	8	Cross-entropy	-
UNet	14,326,275	1 × 10^−5^	0.9	Nadam	16	BCE	0.5
Attention-UNet	8,135,745	1 × 10^−5^	0.9	Nadam	16	BCE	0.5
ResUNet	1,048,953	1 × 10^−5^	0.9	Nadam	16	BCE	0.5
ResUNet++	16,228,001	1 × 10^−5^	0.9	Nadam	16	BCE	0.5
IRv2-Net (Proposed)	28,864,481	1 × 10^−5^	0.9	Nadam	16	BCE	0.5

**Table 3:** Result assessment on Kvasir-SEG dataset where (-) denotes the null value and bold number denotes the highest score.

Method	Accuracy	DSC	IoU	Recall	Precision
NanoNet-A [16]	0.9456	0.8227	0.7282	0.8588	0.8367
UNeXt [17]	-	0.7318	0.6284	0.8843	0.9043
TransNetR [18]	-	0.8706	0.8016	0.8843	0.9073
FCN+CNN [19]	-	**0.8922**	0.8022	-	-
DeepLabv3+ with ResNet50 [49]	-	0.8572	0.7759	0.8616	0.8907
DeepLabv3+ with ResNet101 [49]	-	0.8643	0.7862	0.8592	0.9064
DoubleUNet [50]	-	0.8129	0.7332	0.8402	0.8611
DDANet [51]	-	0.8576	0.7800	0.8880	0.8643
ColonSegNet [52]	0.9493	0.8206	0.7239	0.8496	0.8435
UACANet [53]	-	0.8502	0.7692	0.8799	0.8706
UNet	0.9382	0.7721	0.7792	0.7249	0.8756
UNet + TTA	0.9391	0.7922	0.7787	0.7096	0.8965
Attention-UNet	0.9485	0.8009	0.7938	0.7758	0.8956
Attention-UNet + TTA	0.9515	0.8386	0.8061	0.7705	0.9200
ResUNet	0.9457	0.7865	0.7926	0.7729	0.8804
ResUNet + TTA	0.9499	0.8344	0.8080	0.7709	0.9092
ResUNet++	0.9457	0.7997	0.7956	0.7083	0.9464
ResUNet++ + TTA	0.9487	0.8196	0.8038	0.7126	**0.9645**
IRv2-Net (Proposed)	0.9674	0.8634	0.8399	**0.8997**	0.9010
IRv2-Net + TTA (Proposed)	**0.9691**	0.8696	**0.8460**	0.8919	0.9171

Bold values indicate the best results.

**Table 4:** Result assessment on CVC-ClinicDB dataset where (-) denotes the missing value and bold number denotes the highest score.

Method	Accuracy	DSC	IoU	Recall	Precision
CaraNet [26]	-	0.9360	0.8870	-	-
Double Attention ResUNet [27]	-	0.8300	-	-	-
PSNet [28]	-	0.928	0.879	-	-
Multiple Encoder–decoder [30]	0.9840	0.8890	0.8935	-	-
HarDNet-MSEG [54]	-	0.9320	0.8820	-	-
TransUNet [55]	-	0.9350	0.8870	-	-
MSPB + CNN [56]	-	0.8130	-	0.7860	0.8090
PraNet [57]	-	0.8980	0.8400	-	-
SIFT [58]	0.984	0.889	0.8935	-	-
Dilated ResFCN [59]	-	0.7900	-	0.8100	0.8100
UNet	0.9528	0.7781	0.7736	0.8425	0.7488
UNet + TTA	0.9605	0.8161	0.7862	0.8154	0.8167
Attention-UNet	0.9678	0.8445	0.8316	0.7534	0.9338
Attention-UNet + TTA	0.9654	0.8164	0.8212	0.7171	0.9476
ResUNet	0.9726	0.8707	0.8529	0.8022	0.9334
ResUNet + TTA	0.9698	0.8434	0.8401	0.7578	0.9507
ResUNet++	0.9728	0.8629	0.8519	0.8033	0.9343
ResUNet++ + TTA	0.9713	0.8413	0.8492	0.7622	0.9388
IRv2-Net (Proposed)	0.9850	0.9338	0.8917	0.9138	0.9442
IRv2-Net + TTA (Proposed)	**0.9855**	**0.9375**	**0.8949**	**0.9151**	**0.9482**

Bold values indicate the best results.

**Table 5:** Cross-dataset evaluation utilizing CVC-ClinicDB, where Kvasir-SEG is used to train the models.

Method	Accuracy	DSC	IoU	Recall	Precision
UNet	0.8722	0.3004	0.5389	0.3419	0.3910
UNet + TTA	0.8813	0.3419	0.5665	0.4001	0.4006
Attention-UNet	0.9158	0.4818	0.6306	0.4495	0.6576
Attention-UNet + TTA	**0.9163**	0.4979	0.6443	0.4855	0.6489
ResUNet	0.9052	0.4584	0.6173	0.4518	0.5744
ResUNet + TTA	0.9058	0.4592	0.6320	0.4606	0.5462
ResUNet++	0.9144	0.4006	0.6081	0.3594	**0.6967**
ResUNet++ + TTA	0.9152	0.3833	0.6092	0.3405	0.5318
IRv2-Net (Proposed)	0.8528	0.4926	0.6041	0.6142	0.3840
IRv2-Net+TTA (Proposed)	0.8738	**0.5469**	**0.6457**	**0.6634**	0.4415

Bold values indicate the best results.

**Table 6:** Cross-dataset evaluation utilizing Kvasir-SEG, where CVC-ClinicDB is used to train the models.

Method	Accuracy	DSC	IoU	Recall	Precision
UNet	0.8863	0.6110	0.6606	0.6633	0.6492
UNet + TTA	0.8962	0.6694	0.7034	**0.7482**	0.6348
Attention-UNet	0.8947	0.6019	0.6703	0.5183	0.7624
Attention-UNet + TTA	0.8951	0.6356	0.6973	0.6204	0.7447
ResUNet	0.8883	0.5718	0.6631	0.4937	0.7367
ResUNet + TTA	0.8892	0.5705	0.6731	0.5623	0.6836
ResUNet++	0.8923	0.5646	0.6631	0.4721	0.7839
ResUNet++ + TTA	0.9175	0.6119	0.7002	0.5924	0.7540
IRv2-Net (Proposed)	**0.9369**	**0.7855**	**0.7918**	0.6966	0.8946
IRv2-Net+TTA (Proposed)	0.9355	0.7713	0.7876	0.6710	**0.9112**

Bold values indicate the best results.

## Data Availability

The pre-processed dataset used in this study can be made available with reasonable request to corresponding author.
